# Potency of Selected Berries, Grapes, and Citrus Fruit as Neuroprotective Agents

**DOI:** 10.1155/2020/3582947

**Published:** 2020-05-30

**Authors:** Shih Jen Tan, Intan Safinar Ismail

**Affiliations:** ^1^Laboratory of Natural Products, Institute of Bioscience, Universiti Putra Malaysia, 43000 Serdang, Selangor, Malaysia; ^2^Department of Chemistry, Faculty of Science, Universiti Putra Malaysia, 43400 Serdang, Selangor, Malaysia

## Abstract

A healthy diet should nourish the brain with essential nutrients, including bioactive compounds, for normal brain functioning and to protect it from the negative effects of inflammation and oxidative stress. In this review, a concise summation of the protective effects of selected fruits, namely, berries, grapes, and citrus fruits, against neurological disorder is presented. The focus is on the neuroprotective potential of these fruits against neurodegenerative and mental disorders. The fruits selection was based on the vast reported pharmacological studies on their neuroprotection efficacies. Hence, the respective knowledge and limitations are discussed based on the biological and pharmacological evidence compiled from the previously reported laboratory, epidemiology, and intervention trials.

## 1. Introduction

### 1.1. An Overview on the Global Prevalence of Neurodegenerative Disorders

Cognition is a cluster of brain functions which involves memory, speech, language, reasoning, judgement, planning, learning, compassion, and other thinking abilities. A decline in cognitive abilities is a common aspect of the normal aging process. However, in some instances, severe cognitive impairment occurs as a result of neurological disorders, including various forms of dementia [1]. The global prevalence of dementia is predicted to be 115 million by 2050 based on an expectation of doubling every 20 years from an estimated 36 million in 2010 [2]. Dementia can be classified into a few subtypes, namely, Alzheimer's disease (AD), vascular dementia, dementia with Lewy bodies, frontotemporal dementia, and Creutzfeldt-Jakob disease [3]. Alzheimer's disease is the most common type of dementia, accounting for 60–80% of cases. Alzheimer's disease is reported as the 6th leading cause of death in the United States, where about 5.4 million Americans have AD, with an estimate that 700,000 Americans aged ≥65 years would die of this disease in 2016 [3].

The second most prevalent neurodegenerative disorder is Parkinson's disease (PD). Meta-analysis of worldwide data revealed a rising incidence of PD, with more cases observed in North America, Europe, and Australia, compared with Asian countries [4]. However, it is expected that the occurrence of PD in Asia (China, Japan, India, Indonesia, Pakistan, and Bangladesh) will increase twofold between 2005 and 2030 [5]. Similarly, the most common demyelinating disease, multiple sclerosis (MS), has a higher incidence in the developed countries of North America and Europe than in developing areas such as Eastern Asia and Sub-Saharan Africa [6]. In Malaysia, about 14.3% of the ageing population has been estimated to have dementia. Malay and Bumiputera ethnic groups have a higher incidence rate than other ethnic backgrounds [7]. With respect to cognitive impairment, a higher number of cases has been reported among the Malaysian elderly living in rural communities (22.4%) compared with the urban areas (4%) [8, 9].

In addition to neurodegenerative diseases, mental illness also contributed significantly to the 37.6% global growth in the healthcare burden of brain disorders from 1990 to 2010 [10]. According to a study conducted by Kessler et al. [11], adults from Western countries were more prone to acquire mental illnesses compared to Asians (18% for Chinese and 24% for Japanese). The populations in the United States of America (USA) (55.3%) and France (47.2%) have a higher projected lifetime risk of any mental disorders. Over a third of the total European population (38.2%) also suffers from mental disorders [12].

Although depression is less common among the community-dwelling elderly in Malaysia (7.6%), a critical rate of depression cases has been reported among adults in elder care centres (67%), which is about twice as high as the figure reported for the United Kingdom [8, 13]. In contrast to the elderly population, Malaysian young adults are facing a different set of mental health problems. Anxiety has been documented as the dominant psychological problem among Malaysian undergraduate students, followed by depression and stress. It is especially worth noting that young Malaysian adults (aged 16–24) had the highest incidence of acute and chronic suicidal ideation compared to other age groups [14]. The occurrence of mental health issues among children and adolescents in Malaysia aged five to fifteen years is also a rising trend, increasing from 13% in 1996 to 20% in 2011 [15].

The overall scenario of increasing neurodegenerative diseases and mental disorders is a worrying trend. With the global incidence increase related to neurodegenerative diseases, the socioeconomic burden is also rising for healthcare systems throughout the world, resulting in a negative effect on familial interactions and work productivity. Despite the availability of drugs for combating some of these disease states, the drugs are typically for symptomatic treatment and are associated with contradictory indications and side effects for the patients. In some countries, where approval has been received, cognitive symptoms of AD are treated with cholinesterase inhibitors, such as donepezil, rivastigmine, and galantamine [16], whereas a formulation of levodopa and carbidopa is frequently prescribed for dopaminergic PD therapy [17, 18]. Riluzole, an antiglutamate agent, is the only US Food and Drug Administration-approved medication prescribed for Amyotrophic Lateral Sclerosis (ALS) [19]. Several disease-modifying agents, such as interferon beta-1*β*, interferon beta-1*α*, and glatiramer acetate are widely used to reduce the exacerbation of multiple sclerosis (MS) [20]. However, side effects, including nausea, vomiting, diarrhea, dizziness, weight loss, and motor complications, and adverse effects on the cardiovascular system, make the currently available medications unsatisfactory for complete treatment of the diverse neurodegenerative disorders [21–24].

The discovery and development of medicinal agents from nature for treating neurodegenerative disorders remains one of the most important challenges in the healthcare sector at a time, when populations are ageing significantly throughout the world, as life expectancy steadily increases. Fortunately, the impressive chemical, and thus biological, diversity of ecosystems provides a vast pool of bioactive compounds constituting a myriad of potentially useful medicinal agents to be explored [25]. The expanding field of nutritional neuroscience and nutrigenomics with regard to the prevention or treatment of disease is dedicated to the study of the effects of food and human nutrition on brain plasticity, functions, behaviours, and disease mechanisms. The therapeutic potential of selected natural products, such as fruits, in neuropsychologic conditions, including neurodegenerative and mental disorders, merits detailed studies. In our paper, knowledge of the neuroprotective effects of fruits is discussed.

## 2. Mediators of Inflammation and Antioxidant Biochemistry

Inflammation is a normally controlled and self-limited protective response involving host cell, blood vessel, and proteins which are primarily triggered to eliminate the initiating cause of cell injury to remove necrotic cells and tissue and to promote the repair process [26]. However, an inappropriate inflammatory response may be induced by chemical mediators produced by a damaged host cell due to variety of cues, including infection, traumatic injury, toxic metabolites, or autoimmunity [27]. A complex inflammatory response of an acute brain injury or infection involves activation of microglia, astrocytes, and increased production of cytokines, chemokines, antibodies, and T-lymphocytes [28]. A chronic neuroinflammation is due to a prolonged activation of microglia, a source of multiple neurotoxic factors, including tumour necrosis factor-*α* (TNF-*α*), nitric oxide (NO), interleukin-1*β* (IL-1*β*), and reactive oxygen species (ROS), leading to a progressive neuronal death [27]. Pro- and anti-inflammatory cytokines are characterized based on their structural homology or receptors, in which the activation of receptor triggered when a cytokine ligand bound to its cognate receptor cascading various signals events in cells include activation, adhesion, phagocytosis, cytokine secretion, proliferation, survival, death, apoptosis, and angiogenesis [29, 30]. Anti-inflammatory agents possess the capacity to inhibit inflammation-associated factors and cytokines production (nitric oxide (NO), IL-1*β*, IL-6, IL-10, and TNF-*α*) along with gene downregulation of iNOS and IL-1*β* and through the suppressing of nuclear factor-*κ*B (NF-*κ*B) and activator protein-1 (AP-1) activation besides protein kinases, for example, mitogen-activated protein kinases (MAPK) and protein kinase C (PKC) [31, 32]; Chiou et al., 2000). Nitric oxide is a common focused cytokine when inflammation is investigated. It is a versatile free radical; it triggers a series of inflammatory responses and plays a pivotal role in neurotransmission, vasodilation, and immune regulation in a multiplicity of tissues at physiological concentrations [32].

Antioxidants much associated with having anti-inflammatory properties are exogenous/endogenous molecules that mitigate any form of oxidative/nitrosative stress [33, 34]. The oxidants/antioxidants are organic molecules or enzymes, in which their disequilibrium leads to oxidative or nitrosative stress that mediated in the etiology of numerous human diseases [35]. The common biomarkers of oxidative/nitrosative stress damage, resulting from the lipid peroxidation, are variety of relatively stable decomposition end products or free radicals such as reactive oxygen species (ROS) [34]. The examples of free radicals are hydroxyl free radical, superoxide free radical anion, lipid peroxyl, lipid peroxide, and lipid alkoxyl, and ROS derivatives such as singlet oxygen and hydrogen peroxide [33]. Natural compounds have been reported in numerous preclinical studies as antioxidants having anti-inflammatory properties. Flavonoids and phenolic compounds are among the famous natural antioxidants shown to have anti-inflammatory candidates. Trace metals such as Cu, Zn, Mg, Mn, and Se are also known to have similar bioproperties [36]. These compounds' and elements' biological antioxidative mechanism is mainly by blocking the main production of various proinflammatory mediators via the two major signalling pathways NF-*κ*B and mitogen-activated protein kinases (MAPK) [33]. However, the antioxidants activity depends on a specific set of conditions, with particular importance, which are their dosage and redox conditions in the cell. In this review, the data discussed are based on the reported studies on the selected fruits consisting of antioxidative constituents in specified treatment conditions as having possible anti-inflammatory properties against the neurodegenerative illnesses. Nevertheless, the clinical role of dietary antioxidants or antioxidant supplements such as fruits and vegetables in preventing the diseases associated with oxidative stress still remains uncertain [34].

## 3. The Neuroprotective Potency of Fruits

The health benefits of plant-based foods, especially vegetables and fruits, are undeniable. In addition to their dietary fibre, vitamin, and mineral contents, plant-based foods are well-established as a major source of dietary antioxidants [37]. On average, an American adult consumes 0.5 gram of dietary antioxidants per day from the polyphenols of fruits and vegetables at about 255 and 218 mg/day, respectively [38, 39]. Dietary antioxidants provide a vital cellular protective function against the destructive effects of oxidative free radicals, which have been implicated in the acceleration of ageing and degenerative and chronic diseases [35, 40]. Komatsu et al. [41] significantly related (*P* < 0.001) the high oxidative stress and lower average lifespan of Mongolians to their dietary pattern, which was generally high in meat, flour, and dairy products, but low in fruits and vegetables. Meta-analysis of the relationship between dietary pattern and depression indicated that a ‘healthy' diet, according to current dietary guidelines, includes a recommendation of a high intake of fruit, vegetables, whole grains, fish, poultry, and reduced-fat dairy products and was strongly associated (*P* < 0.001) with a reduced incidence of depression [42]. A positive correlation (*P* < 0.05) between the high consumption of fruits and vegetables (up to 500 g/day) with cognitive performance was also reported [43]. Drinking fruit or vegetable juices at least three times per week also has been linked (*P* < 0.01) to a lower risk of AD [44]. An adjunctive dietary intervention trial targeted the enhancement of dietary quality by encouraging the consumption of foods such as fruits, vegetables, and nuts, which supported an efficacious strategy (*P* < 0.001) for treating major depression [45].

### 3.1. Berries

Berries, such as *Vaccinium cyanococcus* (blueberries), *Rubus fruticosus* (blackberries), *Rubus idaeus* (raspberries), and *Vaccinium macrocarpon* (cranberries), etc., are a group of small, pulpy, and brightly coloured fruits which are consumed worldwide in either a raw or processed form. Recent epidemiological findings indicated that the consumption of anthocyanin-rich foods, such as berries, was associated with lowered risk of PD [46]. This is in line with previous observations, wherein a diet enriched in berries showed cellular protection against oxidative stress through improvement of the motor and cognitive performance in aged mice [47, 48]. Kim et al. [49] established that 70% ethanol extract of mulberry (*Morus alba* L.) fruit was effective against 6-hydroxydopamine (6-OHDA) oxidative stress-induced apoptosis in a human neuroblastoma cell line and reversed 6-OHDA and 1-methyl-4-phenylpyridinium (MPP^+^) neurotoxicity in primary rat dopaminergic neuron cultures. In addition, mulberry extract was active in a murine PD model against 1-methyl-4-phenyl-1,2,3,6-tetrahydropyridine- (MPTP-) induced motor impairment and dopaminergic neuronal loss. The therapeutic potential in neurological disorders was also observed in *Vaccinium cyanococcus* (blueberry) extract, which regulated the expression of inducible nitric oxide synthase (iNOS) and cyclooxygenase-2 (COX-2), at the transcriptional level in BV2 microglial cells on lipopolysaccharides (LPS) assault [50]. *Rubus idaeus* (raspberry) extract was observed to provide an inhibitory effect (*P* < 0.05) on peroxynitrite-mediated hydroxyl radical formation and DNA damage in primary rat cortical astrocyte cultures [51].

The attractive appearance of berries is mainly contributed to by the presence of anthocyanins, a group of pigments, providing the bright red orange to blue violet colours of many fruits and vegetables. Anthocyanins are a flavonoid subgroup and occur naturally in the form of glycosides, in which an anthocyanidin scaffold is conjugated with a sugar. The powerful antioxidant properties of fruits, such as berries, at a suitable determined amount contribute to combating various oxidative stress-related disorders. A comparative study between strawberries with banana and orange carried out by Heo and Lee [52] disclosed that *Fragaria x ananassa* (strawberries) possessed a relatively higher anthocyanin content (19.4 mg of cyanidin 3-glucoside/100 g of fresh weight) than the other two fruits and provided the highest antiapoptotic effect in PC12 cells under oxidative stress. The oxidative stress stimulated by rotenone-induced mitochondrial dysfunction in rat primary dopaminergic neurons culture was greatly attenuated (*P* < 0.05) by an anthocyanin and proanthocyanin rich extract of blueberries when compared with control without rotenone or extract [53]. A significant reduction in *β*-amyloid (A*β*) peptide production, improvement in cognitive performance, and the upregulation of cerebral antioxidant enzyme activities (glutathione *S*-transferase and catalase), along with elevated nuclear factor (erythroid-derived 2)-like 2 (Nrf2) activities in SAMP8 mice, was documented when the subjects were fed a diet supplemented with an anthocyanin-rich mulberry extract [54].

The immunomodulating properties via inhibition of LPS-induced nitrite production of berries have been correlated (*P* < 0.001) with their anthocyanin content. *Rubus fruticosus* (blackberry) anthocyanin extract, which is predominantly comprised of cyanidin-3-*O*-glucoside, suppressed nuclear factor-*κ*B- (NF-*κ*B-) mediated nitric oxide production [55] and alleviated the inflammatory events, including neutrophil infiltration, due to edema induced by carrageenan, lipid peroxidation, nitrosative stress, and prostaglandin E_2_ (PGE_2_) production in a murine pleurisy model [56]. Recent findings demonstrated the suppressive effect of cyanidin-3-*O*-glucoside on the translocation of toll-like receptor 4 (TLR4) to the membrane lipid raft, which suggested its role in restricting TLR4-mediated inflammatory responses [57]. Cyanidin-3-*O*-glucoside is reported to have a neuroprotective effect in mice with focal cerebral ischemia [58]. This anthocyanin was also found to exert a protective effect against A*β-*peptide-induced cognitive impairment and downregulated the A*β*-activated glycogen synthase kinase 3*β* (GSK-3*β*) and tau phosphorylation in a laboratory mouse [59].

The neuromodulatory properties of berries might be attributed to their regulation of several active enzymes in the central nervous system (CNS) by suppressing monoamine oxidase (MAO). Monoamine oxidase has a vital role in maintaining the homeostatic level of amine neurotransmitters in the CNS. Two isoenzymes of MAO (MAO-A and MAO-B) are distributed unevenly in the human brain, wherein their main functions in the CNS are inactivation of the neurotransmitters through catalysis of the oxidative deamination of serotonin, dopamine, histamine, norepinephrine, and epinephrine. MAO inhibitors have been used as antidepressants for decades and it was recently suggested that they might possess therapeutic effectiveness for other neurological disorders, such as Parkinson's disease (PD) and Alzheimer's disease (AD) [65]. An in vitro study indicated that berry anthocyanin showed strong inhibitory effects on both monoamine oxidase A (*P* < 0.0001) and B (*P*=0.0004) enzyme activity [66]. In addition, blueberry anthocyanins improved (*P* < 0.05) cognitive function in rodents after trimethyltin- (TMT-) induced neurotoxicity through inhibition of acetylcholinesterase activity and lipid peroxidation [67]. Restoring brain acetylcholine levels by inhibiting acetylcholinesterase activity is one of the well-established actions exhibited by numerous constituents of functional foods in their preventative roles related to cognitive deterioration [63–66].

Berry consumption might also be beneficial for the enhancement of cognitive abilities. Clinical studies revealed that a 12 week daily consumption of wild blueberry juice significantly improved learning and memory performance among the elderly [67]. Epidemiological findings also revealed weak association (*P* ≥ 0.8) between a greater intake of blueberries and strawberries and a slower progression of cognitive decline in elder women [68]. In animal studies, lyophilised *Vaccinium* spp. (blueberry and bilberry) orally administered for 30 days augmented (*P* < 0.05) the working and short-term memory [69]. Biochemical analysis suggested the improvement of spatial memory performance in aged rodents fed with a blueberry diet was mediated through activation of the ERK-CREB-BDNF pathway [70]. Additionally, the effects of berry intake in minimizing cerebral oxidative stress and inflammatory responses, as well as the regulation of acetylcholinesterase are considered as elementary mechanisms to protect against aged-related cognitive decline [71, 72]. Localization of anthocyanins in several brain regions, including the cerebellum, cortex, hippocampus, and striatum, (*P* < 0.05) after an eight-week, blueberry-supplemented diet, was attributed by Andres-Lacueva et al. [73] to these berry metabolites. Collectively, these findings provide significant fundamental pharmacological and preliminary clinical evidence that reinforces the beneficial influence of berry intake for overall brain health and cognitive performance.

Ellagic acid (2,3,7,8-tetrahydroxybenzopyrano [5,4,3-cde]benzopyran-5,10-dione) is a polyphenolic compound present in various berries, which was reported to modulate significantly inflammatory events in in vitro and in vivo [74–76]. This compound was active in affording protection against A*β*-induced neurotoxicity in neuroblastoma SH-SY5Y cells [77]. Oral administration of ellagic acid in the murine Alzheimer's disease model before scopolamine assault was found to significantly ameliorate lipid peroxidation and brain endogenous antioxidant capacity, thereby improving motor and cognitive performance [78]. The reported activity of ellagic acid on *β-*site APP-cleaving enzyme 1 (BACE1) inhibition underlines its regulatory role in APP cleavage and A*β* secretion [79]. Ellagic acid was also effective in preventing inflammatory responses and motor and cognitive impairments in long-term potentiation impairments induced by traumatic brain injury (TBI) and in a 6-OHDA-induced Parkinsonian mouse model [80, 81]. Oxidative stress in an animal model of diabetic neuropathy was ameliorated significantly by ellagic acid [82]. Apart from neurodegenerative disorders, several studies have reported the potency of ellagic acid against major depression by emphasizing its antidepressant activities on the modulation of monoamine neurotransmitter systems in in vivo models [83, 84].

### 3.2. Grape (*Vitis vinifera*)

Grapes (*Vitis vinifera*) can be categorized as a great source of natural neurotrophic agents [85]. Similar to berries, grapes are also a concentrated source of anthocyanins, which makes the grape skins red, purple, black, or green. Intact anthocyanin molecules could be detected in plasma and brain samples after a grape anthocyanin mixture was given to rats [86]. The bioactive chemicals of grapes are present in the skin and edible fruit pulp and in grape seed extract which was reported to enhance the antioxidant status through upregulation of the endogenous antioxidant enzyme activities, including glutathione peroxidase, catalase and superoxide dismutase, and nonenzymatic antioxidant levels, followed by the decrease in radical-induced lipid peroxidation in the CNS of aged rats [87]. The major antioxidant in grapes is attributed to resveratrol, besides the phenolic compounds such as gallic acid, ellagic acid, and their derivatives, epigallocatechin gallate, epicatechin gallate, epigallocatechin, epicatechin, catechin, and their derivatives [88, 89]. Oral administration of grape seed polyphenolic extract inhibited A*β* peptide aggregation and oligomerization and attenuated cognitive deterioration in Tg2576 mice. The A*β*-soluble high-molecular weight oligomers have been proposed to be highly responsible for Alzheimer's disease [90]. Rotenone-induced dopaminergic neuron degeneration in rat primary midbrain cultures was also reduced following treatment with grape seed extract rich in anthocyanin and proanthocyanin content [53]. Carvalho et al. [91] have reported that grape skin anthocyanins were active in weakening inflammatory events, including neutrophil infiltration, in addition to rescuing the ethidium bromide-induced demyelination of neurons in the pons of rat. The grape leaves extract intake for 12 days by male Sprague Dawley rats suppressed the ethanol-induced expression of nuclear factor-*κ*B (NF-*κ*B) p65 subunit, proinflammatory cytokine tumour necrosis factor-*α*, caspase-3, and surviving [92].

3,4′,5-Trihydroxy-*trans*-stilbene (resveratrol) is a major compound present in grapes [93]. It possesses immunomodulatory effects, which suppresses the proliferation of splenic lymphocytes [94] and regulates the production of PGE_2_ [95] and other proinflammatory markers, such as nitric oxide (NO) and tumour necrosis factor alpha (TNF-*α*) [96]. Similarly, resveratrol significantly decreased the 6-OHDA-induced pro-inflammatory cytokines levels of COX-2 and TNF-a mRNA in the substantia nigra of rodents as detected by real-time PCR (RT-PCR) [97]. Resveratrol has been documented as a potent antioxidant agent. For instance, the inhibition exerted by resveratrol on NADPH oxidase activity and membrane translocation of NADPH oxidase cytosolic p47 subunit effectively diminished the oxidative stress-induced dopaminergic neurodegeneration in the cellular PD model [98]. The antioxidant activity against oxidative stress, i.e., an increase in lipid peroxidation and decrease in the antioxidant glutathione were reproducible in an animal study, where Sharma and Gupta [99] have found that chronic resveratrol treatment alleviated streptozotocin-induced oxidative stress and significantly prevented cognitive impairment in rodents. Khan et al. [100] reported that the same dose of resveratrol (20 mg/kg body weight, i.p.) rescued the oxidative stress-induced neuronal apoptosis through the upregulation of endogenous antioxidant enzyme activity in the mouse brain. Resveratrol was observed to prevent various ailments including degenerative diseases and inflammatory disorders via mechanisms of actions including increase in superoxide dismutase, hemeoxygenase-1, and glutathione peroxidase activities and reduced glutathione content and malondialdehyde (MDA) levels [101].

In addition to the antioxidant and anti-inflammatory properties, the neuroprotective effects of resveratrol might be attributed to intervention in autophagy pathway signalling. Resveratrol was reported to activate the AMP-activated protein kinase (AMPK)/silent information regulator 1 (SIRT1)-mediated autophagy, which in turn enhanced the performance of the protein degradation system in clearing misfolded and aggregated protein [102]. A similar signalling pathway was reported in resveratrol-mediated neuroprotection in an in vitro prion disease model [103]. Moreover, the neurotrophic properties of resveratrol have also been documented, wherein the production of the glia-derived neurotrophic factor (GDNF) and brain-derived neurotrophic factor (BDNF) in primary rat astroglia cultures was induced by resveratrol treatment [104]. Resveratrol treatment was also shown to suppress in vitro microglial-mediated apoptotic neuronal cell death [105] and in vivo MPTP-induced dopaminergic neuronal loss in a mouse PD model [106].

Understanding the mechanisms underlying the pathophysiology of psychiatric disorders remains limited. However, the chemical imbalance of monoamine neurotransmitters, such as serotonin and noradrenaline (monoaminergic hypothesis of depression), was proposed and clinically targeted for the treatment of the symptoms of depression. Previously, resveratrol was suggested to have antidepressant activities through restoration of the monoaminergic functions, specifically acting through normalizing serotonin and noradrenaline levels and regulating the activities of monoamine oxidase-A (MAO-A) [107]. Additionally, the antidepressant activities of resveratrol were repeated in different animal models including a Wistar Kyoto rat model of depression, corticosterone, and chronic unpredictable mild stress- (CUMS-) induced depression [108]. Studies showed that the brain-derived neurotrophic factor and cAMP-response element binding (CREB) protein levels were significantly elevated after the administration of resveratrol in the hippocampus, amygdala, and prefrontal cortex of depressed animals [108–110]. The behavioural abnormalities and elevation of serum corticosterone levels were used for determination of depression in the rat model [110]. In fact, upregulation of the CREB/BDNF pathway was known as the most promising antidepressive mechanism elicited by numerous established antidepressants and phytocompounds in fighting against the symptoms of depression [111–113]. Furthermore, the bioactivity of resveratrol in reducing the cerebral infarct volume and middle cerebral artery occlusion- (MCAO-) induced series of abnormalities in male Wistar rats, including both depressive-like behaviours and biochemical measurements, encouraged additional studies to examine its potential in handling poststroke depression (PSD). Poststroke depression is the most frequent neuropsychiatric consequence of stroke, accounting for 30% recurrence among stroke survivors [114, 115].

### 3.3. Citrus Fruit (*Citrus* spp.)

Citrus fruits in this section refer to the fruits produced from plants in the genus *Citrus*. Examples including clementines (*Citrus clementine*), grapefruits (*C. paradisi*), lemons (*C. limon*), limes (*C. aurantifolia*), oranges (*C. sinensis*), and tangerines (*C. reticulata*) are some of the most commonly eaten citrus fruits around the world. The health benefits of citrus fruits are often ascribed to its outstanding vitamin C content. However, citrus fruits do have other metabolites which are active in various disease interventions, including neurological disorders [116]. In the LPS-activated BV2 microglia culture system, 3′,4′,5,6,7,8-hexamethoxyflavone (nobiletin), a typical citrus flavonoid, was reported to exert similar antineuroinflammation activities (nitric oxide and TNF-*α* productions) to minocycline. The antineuroinflammatory activities were believed to occur through suppression of the NF-*κ*B pathway [117]. An in vitro comparative study among eight tangerine peel flavonoids highlighted that nobiletin was the most effective in attenuating both mRNA and protein expression of proinflammatory markers [118]. Nobiletin was active in vitro and in vivo, whereby nobiletin treatment was found to reduce memory impairment and cholinergic neurodegeneration in olfactory bulbectomized mice [119]. In a transgenic AD mouse model (APP-SL 7-5), nobiletin treatment was able to reduce A*β* oligomer deposition and diminish memory impairment [120]. Moreover, reduction of phosphorylated tau protein levels in the brains of senescence-accelerated mouse prone 8 (SAMP8) was observed in a nobiletin-treated group at 10 or 50 mg/kg [121].

4′,5,7-Trihydroxyflavanone-7-rhamnoglucoside (naringin) is another ubiquitous citrus flavonoid with anti-inflammatory properties. It was reported to regulate the NF-*κ*B signalling pathway through suppression of the degradation of IĸB-*α* and the nucleus translocation of p65 [122]. Antiapoptotic activity of naringin in cellular human SH-SY5Y cells of a Parkinson's disease (PD) model was also observed [123]. Naringin was effective against PD neurotoxicity induced by both 6-OHDA and MPP^+^ through the upregulation of the glia-derived neurotrophic factor (GDNF) in dopaminergic neurons and the suppression of microglia activation in the substania nigra of rodent brains [124, 125]. Apart from this, naringin displayed a significant protective effect against neurotoxin-induced cognitive impairment in rats at 80 mg/kg [126, 127]. Golechha et al. [126] also suggested an antiepileptic activity of naringin.

Dried *Citrus reticulata* (tangerine) peel has been used in traditional Chinese medicine for treating ailments such as bronchial asthma and dyspepsia. In 2014, Ho and Kuo indicated that the antineuroinflammatory activity of tangerine peel was attributed to the collective effect of three citrus flavonoids, which included 3′,5,7-trihydroxy-4′-methoxyflavone-7-rhamnoglucoside (hesperidin). Studies in a murine experimental stroke model revealed that hesperidin possessed a prophylactic effect against brain injury through the upregulation of brain antioxidant capacity, along with the inhibition of the synthesis of proinflammatory markers [128]. Hesperidin showed clinical effects against diabetic neuropathies [129]. Another flavonoid isolated from the citrus peel, 4′,5,6,7,8-pentamethoxy flavone (tangeretin) exhibited in vitro antineuroinflammation by regulating activation of mitogen-associated protein kinases (MAPKs) and the NF-*κ*B pathways in microglial cells. These activities resulted in downregulation of various proinflammatory mediators, such as cytokines and inflammation-related enzymes [130].

7-[(2E)-3,7-Dimethylocta-2,6-dienoxy]chromen-2-one (auraptene) is a coumarin derivative, which has been reported to possess various pharmacological properties including neuroprotective effects [131] on the mouse brain by suppressing inflammation, e.g., through reduction in hyperactivation of microglia and astrocytes [132]. The anti-inflammatory effects were not only on the peripheral organs but also the brain of a mouse model of global cerebral ischemia [133, 134] and lipopolysaccharide- (LPS-) induced systemic inflammation [135, 136]. The induction of the brain-derived neurotrophic factor (BDNF) in neuronal cells was suggested to be one of the mechanisms accounting for the neuroprotective effects exerted by AUR. The BDNF mRNA levels and secreted BDNF in neuro-2a cells were significantly increased by AUR in a dose- and time-dependent manner [132].

## 4. Conclusion and Perspectives

In addition to the impact on the quality of life for the sufferers, families, and caregivers, mental health disorders, as a health category, are one of the heaviest burdens on the world's economy. In 2010, the total European cost of mental healthcare was estimated as €798 billion [137], and a total worldwide cost for dementia care was US $604 billion in the same year, with about 70% of those costs incurred in Western Europe and North America [138]. A healthy brain from the aspect of both mental and cognitive functions is essential for a good quality of life, well-being, and independent living. Aside from the limitations and potential side effects of the current therapeutic approaches for brain disorders, a healthy lifestyle, together with a healthy eating regimen, is strongly recommended as a prophylactic approach for maintaining a vibrant state of health, including brain health. In vitro and in vivo studies have established that fruits and nuts are well endowed with metabolites that are active in modulating the pathogenesis of brain disorders and for improving cognitive behaviours. Compelling epidemiologic observations indicate a strong correlation between a healthy eating pattern and a lower risk of neurological disorders and better mental health.

However, current studies are insufficient to provide more definitive information which relates to optimal servings of these foods or to recommended doses of active phytoconstituents for neuroprotective actions in humans or to interactions with other foods which may act synergistically or antagonistically with prophylactic metabolites. Animal and in vitro studies have shed light on the possible metabolism-based therapies for the mental illnesses, as well as aging, ischemia, trauma, and mitochondrial cytopathies. However, the clinical investigation on the consumption of the selected fruits, which are efficacious as neuroprotective agents, is important. Evidence from larger clinical cohorts and longer trial periods under well-defined controlled conditions is necessary for a more precise estimation of the neuroprotective effects of functional foods, including *Citrus* spp., grapes, nuts, and berries, in neuronal and brain disorders (Table 1). In addition, there remains a missing piece of information, namely, regarding the therapeutic efficacy of these functional foods in the patients already suffering from neurodegenerative and mental illness. A well-coordinated and multidisciplinary effort between the neurosciences and the nutritional sciences using standardized preparations is expected in the future to provide recommendations and guidelines for safe and effective dietary interventions for brain health and protective effects against neurological and brain disorders.

Due to the complexity between the nutritional effects of the foods and human health, the present study recommends that the consumption of fruits and nuts based on the guidelines of US Food and Drug Administration [139] on a daily basis (Code of Federal Regulations, Title 21; April 1, 2018). Fruits and nuts are affordable snacks and easily incorporated as part of a balanced diet.

## Additional Points

*Summary.* Chemical compounds studied in this article are as follows: 2-phenyl-2-[3,4,5-trihydroxy-6-[[3,4,5-trihydroxy-6-(hydroxymethyl)oxan-2-yl]oxymethyl]oxan-2-yl]oxyacetonitrile (amygdalin) (PubChem CID: 656516); 17-(5-ethyl-6-methylheptan-2-yl)-10,13-dimethyl-2,3,4,7,8,9,11,12,14,15,16,17-dodecahydro-1H-cyclopenta[a]phenanthren-3-ol (*β*-sitosterol) (PubChem CID: 222284); (3S,8S,9S,10R,13R,14S,17R)-17-[(2R,5R)-5,6-dimethylheptan-2-yl]-10,13-dimethyl-2,3,4,7,8,9,11,12,14,15,16,17-dodecahydro-1H-cyclopenta[a]phenanthren-3-ol (campesterol) (PubChem CID: 173183); 6,7,13,14-tetrahydroxy-2,9-dioxatetracyclo[6.6.2.04,16.011,15]hexadeca-1(15),4,6,8(16),11,13-hexaene-3,10-dione (ellagic acid) (PubChem CID: 5281855); (2S)-5-hydroxy-2-(3-hydroxy-4-methoxyphenyl)-7-[(2S,3R,4S,5S,6R)-3,4,5-trihydroxy-6-{[(2R,3R,4R,5R,6S)-3,4,5-trihydroxy-6-methyloxan-2-yl]oxymethyl}oxan-2-yl]oxy-2,3-dihydrochromen-4-one (hesperidin) (PubChem CID: 10621); 7-[[2-O-(6-deoxy-*α*-L-mannopyranosyl)-*β*-D-glucopyranosyl]oxy]-2,3-dihydro-5-hydroxy-2-(4-hydroxyphenyl)-4H-1-benzopyran-4-one (naringin) (PubChem CID: 442428); 7-[(2E)-3,7-dimethylocta-2,6-dienoxy]chromen-2-one (auraptene); 2-(3,4-dimethoxyphenyl)-5,6,7,8-tetramethoxychromen-4-one (nobiletin) (PubChem CID: 72344); 5-[(E)-2-(4-hydroxyphenyl)ethenyl]benzene-1,3-diol (resveratrol) (PubChem CID: 445154); (3S,8S,9S,10R,13R,14S,17R)-17-[(E,2R,5S)-5-ethyl-6-methylhept-3-en-2-yl]-10,13-dimethyl-2,3,4,7,8,9,11,12,14,15,16,17-dodecahydro-1H-cyclopenta[a]phenanthren-3-ol (stigmasterol) (PubChem CID: 5280794); 5,6,7,8-tetramethoxy-2-(4-methoxyphenyl)-4H-1-benzopyran-4-one (tangeretin) (PubChem CID: 68077).

## Abbreviations

AP-1: Activator protein-1
AD: Alzheimer's disease
AMPK: AMP-activated protein kinase
BDNF: Brain-derived neurotrophic factor
CNS: Central nervous system
COX-2: Cyclooxygenase-2
GDNF: Glia-derived neurotrophic factor
6-OHDA: 6-Hydroxydopamine
LPS: Lipopolysaccharides
MAPK: Mitogen-activated protein kinases
MPP^+^: Methyl-4-phenylpyridinium
MPTP: 1-Methyl-4-phenyl-1,2,3,6-tetrahydropyridine
MAPKs: Mitogen-associated protein kinases
MAO: Monoamine oxidase
NO: Nitric oxide
iNOS: Nitric oxide synthase
NF-*κ*B: Nuclear factor-*κ*B
PD: Parkinson's disease
PGE_2_: Prostaglandin E_2_
PKC: Protein kinase C
SIRT1: Silent information regulator 1
TLR4: Toll-like receptor 4.

## Supplementary Materials

Supplementary Materials Table 1: summary of the neuroprotective potency of selected fruits.

## References

[1] Harada C. N., Natelson Love M. C., Triebel K. L. (2013). Normal cognitive aging. *Clinics in Geriatric Medicine*.

[2] Prince M., Bryce R., Albanese E., Wimo A., Ribeiro W., Ferri C. P. (2013). The global prevalence of dementia: a systematic review and metaanalysis. *Alzheimer’s & Dementia*.

[3] Association A. (2016). 2016 Alzheimer’s disease facts and figures. *Alzheimer’s & Dementia*.

[4] Pringsheim T., Jette N., Frolkis A., Steeves T. D. L. (2014). The prevalence of Parkinson’s disease: a systematic review and meta-analysis. *Movement Disorders*.

[5] Tan L. C. S. (2013). Epidemiology of Parkinson’s disease. *Neurology Asia*.

[6] Leray E., Moreau T., Fromont A., Edan G. (2016). Epidemiology of multiple sclerosis. *Revue Neurologique*.

[7] Hamid T. A., Krishnaswamy S., Abdullah S. S., Momtaz Y. A. (2010). Sociodemographic risk factors and correlates of dementia in older Malaysians. *Dementia and Geriatric Cognitive Disorders*.

[8] Sidik S. M., Rampal L., Afifi M. (2004). Physical and mental health problems of the elderly in a rural community of Sepang, Selangor. *Malaysian Journal of Medical Science*.

[9] Zainiyah S. S. Y., Gunasegaran M., Muhammad Hanif M. Z., Nurumalina N., Seow H. C., Bharathi V. (2011). Prevalence of cognitive impairment among the members of the National Council of Senior Citizens’ Malaysia in day care centres within the Klang valley. *Malaysian Journal of Public Health Medicine*.

[10] Whiteford H. A., Degenhardt L., Rehm J. (2013). Global burden of disease attributable to mental and substance use disorders: findings from the global burden of disease study 2010. *The Lancet*.

[11] Kessler R. C., Angermeyer M., Anthony J. C. (2007). Lifetime prevalence and age-of-onset distributions of mental disorders in the World Health Organization’s world mental health survey initiative. *World Psychiatry: Official Journal of the World Psychiatric Association (WPA)*.

[12] Wittchen H. U., Jacobi F., Rehm J. (2011). The size and burden of mental disorders and other disorders of the brain in Europe 2010. *European Neuropsychopharmacology*.

[13] Al-Jawad M., Rashid A. K., Narayan K. A. (2007). Prevalence of undetected cognitive impairment and depression in residents of an elderly care home. *Medical Journal of Malaysia*.

[14] Shamsuddin K., Fadzil F., Ismail W. S. W. (2013). Correlates of depression, anxiety and stress among Malaysian university students. *Asian Journal of Psychiatry*.

[15] Ahmad N., MuhdYusoff F., Ratnasingam S. (2015). Trends and factors associated with mental health problems among children and adolescents in Malaysia. *International Journal of Culture and Mental Health*.

[16] Casey D. A., Antimisiaris D., O’Brien J. (2010). Drugs for Alzheimer’s disease: are they effective?. *Pharmacy & Therapeutics*.

[17] Rascol O., Perez-Lloret S., Ferreira J. J. (2015). New treatments for levodopa-induced motor complications. *Movement Disorders,*.

[18] Waters C. H., Nausieda P., Dzyak L. (2015). Long-term treatment with extended-release carbidopa-levodopa (IPX066) in early and advanced Parkinson’s disease: a 9-month open-label extension trial. *CNS Drugs*.

[19] Miller R. G., Mitchell J. D., Moore D. H. (2012). Riluzole for amyotrophic lateral sclerosis (ALS)/motor neuron disease (MND). *Cochrane Database of Systematic Reviews*.

[20] Goldenberg M. M. (2012). Multiple sclerosis review. *P & T: A Peer-Reviewed Journal for Formulary Management*.

[21] Amanzio M., Palermo S., Zibetti M. (2014). Self-unawareness of levodopa induced dyskinesias in patients with Parkinson’s disease. *Brain and Cognition*.

[22] Groeneveld G. J., van der Tweel J. H., Veldink I. (2003). A randomized sequential trial of creatine in amyotrophic lateral sclerosis. *Annals of Neurology*.

[23] Hansen R. A., Gartlehner G., Webb A. P., Morgan L. C., Moore C. G., Jonas D. E. (2008). Efficacy and safety of Donepezil, Galantamine, and Rivastigmine for the treatment of Alzheimer’s disease: a systematic review and meta-analysis. *Journal of Clinical Interventions in Aging*.

[24] Tanaka A., Koga S., Hiramatsu Y. (2009). Donepezil-induced adverse side effects of cardiac rhythm: 2 cases report of atrioventricular block and Torsade de Pointes. *Internal Medicine*.

[25] Newman D. J., Cragg G. M. (2016). Natural products as sources of new drugs from 1981 to 2014. *Journal of Natural Products*.

[26] Azam A. A., Ismail I. S., Shaikh M. F., Shaari K., Abas F. (2019). Effects of Clinacanthus nutans leaf extracts on lipopolysaccharides (LPS)-induced neuroinflammation rats observed via behavioral pattern and ^1^H NMR-based metabolomics approach. *Avicenna Jounal of Phytomedicine*.

[27] Lull M. E., Block M. L. (2010). Microglial activation and chronic neurodegeneration. *Neurotherapeutics*.

[28] Rock R. B., Gekker G., Hu S. (2004). Role of microglia in central nervous system infections. *Clinical Microbiology Reviews*.

[29] Devi L. (2000). G-protein-coupled receptor dimers in the lime light. *Trends in Pharmacological Sciences*.

[30] Walz A., Peveri P., Aschauer H., Baggiolini M. (1987). Purification and amino acid sequencing of NAF, a novel neutrophil-activating factor produced by monocytes. *Biochemical and Biophysical Research Communications*.

[31] de Oliveira C. M. B., Sakata R. K., Issy A. M., Gerola L. R., Salomão R. (2011). Cytokines and pain. *Brazilian Journal of Anesthesiology*.

[32] Chiou W. F., Chen C. F., Lin J. J. (2000). Mechanisms of suppression of inducible nitric oxide synthase (iNOS) expression in RAW 264.7 cells by andrographolide. *British Journal of Pharmacology*.

[33] Arulselvan P., Fard M. T., Tan W. S. (2016). Role of antioxidants and natural products in inflammation. *Oxidative Medicine and Cellular Longevity*.

[34] Stone W. L., Basit H., Mohiuddin S. S. (2020). *Biochemistry, Antioxidants*.

[35] Kurutas E. B. (2016). The importance of antioxidants which play the role in cellular response against oxidative/nitrosative stress: current state. *Nutrition Journal*.

[36] Ravipati A. S., Zhang L., Koyyalamudi S. R. (2012). Antioxidant and anti-inflammatory activities of selected Chinese medicinal plants and their relation with antioxidant content. *BMC Complementary and Alternative Medicine*.

[37] Liu R. H. (2013). Health-promoting components of fruits and vegetables in the diet. *Advances in Nutrition*.

[38] Vinson J. A., Hao Y., Su X., Zubik L. (1998). Phenol antioxidant quantity and quality in foods: vegetables. *Journal of Agricultural and Food Chemistry*.

[39] Vinson J. A., Su X., Zubik L., Bose P. (2001). Phenol antioxidant quantity and quality in foods: fruits. *Journal of Agricultural and Food Chemistry*.

[40] Lobo V., Patil A., Phatak A., Chandra N. (2010). Free radicals, antioxidants and functional foods: impact on human health. *Pharmacognosy Reviews*.

[41] Komatsu F., Kagawa Y., Sakuma M. (2006). Investigation of oxidative stress and dietary habits in Mongolian people, compared to Japanese people. *Nutrition & Metabolism*.

[42] Lai J. S., Hiles S., Bisquera A., Hure A. J., McEvoy M., Attia J. (2014). A systematic review and meta-analysis of dietary patterns and depression in community-dwelling adults. *The American Journal of Clinical Nutrition*.

[43] Nurk E., Refsum H., Drevon C. A. (2010). Cognitive performance among the elderly in relation to the intake of plant foods. The Hordaland health study. *British Journal of Nutrition*.

[44] Dai Q., Borenstein A. R., Wu Y., Jackson J. C., Larson E. B. (2006). Fruit and vegetable juices and Alzheimer’s disease: the Kame project. *The American Journal of Medicine*.

[45] Jacka F. N., O’Neil A., Opie R. (2017). A randomised controlled trial of dietary improvement for adults with major depression (the “SMILES” trial). *BMC Medicine*.

[46] Gao X., Cassidy A., Schwarzschild M. A., Rimm E. B., Ascherio A. (2012). Habitual intake of dietary flavonoids and risk of Parkinson disease. *Neurology*.

[47] Duffy K. B., Spangler E. L., Devan B. D. (2008). A blueberry-enriched diet provides cellular protection against oxidative stress and reduces a kainate-induced learning impairment in rats. *Neurobiology of Aging*.

[48] Shukitt-Hale B., Cheng V., Joseph J. A. (2009). Effects of blackberries on motor and cognitive function in aged rats. *Nutritional Neuroscience*.

[49] Kim H. G., Ju M. S., Shim J. S. (2010). Mulberry fruit protects dopaminergic neurons in toxin-induced Parkinson’s disease models. *British Journal of Nutrition*.

[50] Lau F. C., Bielinski D. F., Joseph J. A. (2007). Inhibitory effects of blueberry extract on the production of inflammatory mediators in lipopolysaccharide-activated BV2 microglia. *Journal of Neuroscience Research*.

[51] Chen W., Su H., Huang Z., Feng L., Nie H. (2012). Neuroprotective effect of raspberry extract by inhibiting peroxynitrite-induced DNA damage and hydroxyl radical formation. *Food Research International*.

[52] Heo H. J., Lee C. Y. (2005). Strawberry and its anthocyanins reduce oxidative stress-induced apoptosis in PC12 cells. *Journal of Agricultural and Food Chemistry*.

[53] Strathearn K. E., Yousef G. G., Grace M. H. (2014). Neuroprotective effects of anthocyanin- and proanthocyanidin-rich extracts in cellular models of Parkinson’s disease. *Brain Research*.

[54] Shih P.-H., Chan Y.-C., Liao J.-W., Wang M.-F., Yen G.-C. (2010). Antioxidant and cognitive promotion effects of anthocyanin-rich mulberry (Morus atropurpurea L.) on senescence-accelerated mice and prevention of Alzheimer’s disease. *The Journal of Nutritional Biochemistry*.

[55] Pergola C., Rossi A., Dugo P., Cuzzocrea S., Sautebin L. (2006). Inhibition of nitric oxide biosynthesis by anthocyanin fraction of blackberry extract. *Nitric Oxide*.

[56] Rossi A., Serraino I., Dugo P. (2012). Protective effects of anthocyanins from blackberry in a rat model of acute lung inflammation. *Free Radical Research*.

[57] Fu Y., Zhou E., Wei Z. (2014). Cyanidin-3-O-*β*-glucoside ameliorates lipopolysaccharide-induced acute lung injury by reducing TLR4 recruitment into lipid rafts. *Biochemical Pharmacology*.

[58] Min J., Yu S.-W., Baek S.-H. (2011). Neuroprotective effect of cyanidin-3-O-glucoside anthocyanin in mice with focal cerebral ischemia. *Neuroscience Letters*.

[59] Qin L., Zhang J., Qin M. (2013). Protective effect of cyanidin 3-O-glucoside on beta-amyloid peptide-induced cognitive impairment in rats. *Neuroscience Letters*.

[60] Youdim M. B. H., Edmondson D., Tipton K. F. (2006). The therapeutic potential of monoamine oxidase inhibitors. *Nature Reviews Neuroscience*.

[61] Dreiseitel A., Korte G., Schreier P. (2009). Berry anthocyanins and their aglycons inhibit monoamine oxidases A and B. *Pharmacological Research*.

[62] Jo Y. N., Jin D. E., Jeong J. H., Kim H. J., Kim D.-O., Heo H. J. (2015). Effect of anthocyanins from rabbit-eye blueberry (Vaccinium virgatum) on cognitive function in mice under trimethyltin-induced neurotoxicity. *Food Science and Biotechnology*.

[63] Batool Z., Sadir S., Liaquat L. (2015). Repeated administration of almonds increases brain acetylcholine levels and enhances memory function in healthy rats while attenuates memory deficits in animal model of amnesia. *Brain Research Bulletin*.

[64] Kulkarni K. S., Kasture S. B., Mengi S. A. (2010). Efficacy study of *Prunus amygdalus* (almond) nuts in scopolamine-induced amnesia in rats. *Indian Journal of Pharmacology*.

[65] Lee B., Park J., Kwon S. (2010). Effect of wild ginseng on scopolamine-induced acetylcholine depletion in the rat hippocampus. *Journal of Pharmacy and Pharmacology*.

[66] Oboh G., Ademiluyi A. O., Akinyemi A. J. (2012). Inhibition of acetylcholinesterase activities and some pro-oxidant induced lipid peroxidation in rat brain by two varieties of ginger (Zingiber officinale). *Experimental and Toxicologic Pathology*.

[67] Krikorian R., Shidler M. D., Nash T. A. (2010). Blueberry supplementation improves memory in older adults. *Journal of Agricultural and Food Chemistry*.

[68] Devore E. E., Kang J. H., Breteler M. M. B., Grodstein F. (2012). Dietary intakes of berries and flavonoids in relation to cognitive decline. *Annals of Neurology*.

[69] Ramirez M., Izquierdo I., Raseira M., Zuanazzi J., Barros D., Henriques A. (2005). Effect of lyophilised berries on memory, anxiety and locomotion in adult rats. *Pharmacological Research*.

[70] Williams C. M., El Mohsen M. A., Vauzour D. (2008). Blueberry-induced changes in spatial working memory correlate with changes in hippocampal CREB phosphorylation and brain-derived neurotrophic factor (BDNF) levels. *Free Radical Biology and Medicine*.

[71] Goyarzu P., Malin D. H., Lau F. C. (2004). Blueberry supplemented diet: effects on object recognition memory and nuclear factor-kappa B levels in aged rats. *Nutritional Neuroscience*.

[72] Wattanathorn J., Muchimapura S., Thukhammee W. (2012). Mulberry fruits protects against age-related cognitive decline. *American Journal of Applied Sciences*.

[73] Andres-Lacueva C., Shukitt-Hale B., Galli R. L., Jauregui O., Lamuela-Raventos R. M., Joseph J. A. (2005). Anthocyanins in aged blueberry-fed rats are found centrally and may enhance memory. *Nutritional Neuroscience*.

[74] Daniel E. M., Krupnick A. S., Heur Y.-H., Blinzler J. A., Nims R. W., Stoner G. D. (1989). Extraction, stability, and quantitation of ellagic acid in various fruits and nuts. *Journal of Food Composition and Analysis*.

[75] Papoutsi Z., Kassi E., Chinou I., Halabalaki M., Skaltsounis L. A., Moutsatsou P. (2008). Walnut extract (Juglans regia L.) and its component ellagic acid exhibit anti-inflammatory activity in human aorta endothelial cells and osteoblastic activity in the cell line KS483. *British Journal of Nutrition*.

[76] Umesalma S., Sudhandiran G. (2010). Differential inhibitory effects of the polyphenol ellagic acid on inflammatory mediators NF-*κ*B, iNOS, COX-2, TNF-*α*, and IL-6 in 1,2-dimethylhydrazine-induced rat colon carcinogenesis. *Basic & Clinical Pharmacology & Toxicology*.

[77] Feng Y., Yang S.-g., Du X.-t. (2009). Ellagic acid promotes A*β*42 fibrillization and inhibits A*β*42-induced neurotoxicity. *Biochemical and Biophysical Research Communications*.

[78] Kaur R., Mehan S., Khanna D., Kalra S. (2015). Ameliorative treatment with ellagic acid in scopolamine induced Alzheimer’s type memory and cognitive dysfunctions in rats. *Austin Journal of Clinical Neurology*.

[79] Kwak H.-M., Jeon S.-Y., Sohng B.-H. (2005). *β*-secretase (BACE1) inhibitors from pomegranate (Punica granatum) husk. *Archives of Pharmacal Research*.

[80] Farbood Y., Sarkaki A., Dianat M., Khodadadi A., Haddad M. K., Mashhadizadeh S. (2015). Ellagic acid prevents cognitive and hippocampal long-term potentiation deficits and brain inflammation in rat with traumatic brain injury. *Life Sciences*.

[81] Farbood Y., Sarkaki A., Dolatshahi M., Mansouri S. M. T., Khodadadi A. (2015). Ellagic acid protects the brain against 6-hydroxydopamine induced neuroinflammation in a rat model of Parkinson’s disease. *Basic and Clinical Neuroscience*.

[82] Uzar E., Alp H., Cevik M. U. (2012). Ellagic acid attenuates oxidative stress on brain and sciatic nerve and improves histopathology of brain in streptozotocin-induced diabetic rats. *Neurological Sciences*.

[83] Dhingra D., Chhillar R. (2012). Antidepressant-like activity of ellagic acid in unstressed and acute immobilization-induced stressed mice. *Pharmacological Reports*.

[84] Girish C., Raj V., Arya J., Balakrishnan S. (2012). Evidence for the involvement of the monoaminergic system, but not the opioid system in the antidepressant-like activity of ellagic acid in mice. *European Journal of Pharmacology*.

[85] Xia E., He X., Li H., Wu S., Li S., Deng G. (2014). Chapter 5—biological activities of polyphenols from grapes. *Polyphenols in Human Health and Disease*.

[86] Passamonti S., Vrhovsek U., Vanzo A., Mattivi F. (2005). Fast access of some grape pigments to the brain. *Journal of Agricultural and Food Chemistry*.

[87] Balu M., Sangeetha P., Haripriya D., Panneerselvam C. (2005). Rejuvenation of antioxidant system in central nervous system of aged rats by grape seed extract. *Neuroscience Letters*.

[88] Rameshrad M., Imenshahidi M., Razavi B. M., Iranshahi M., Hosseinzadeh H. (2018). Bisphenol A vascular toxicity: protective effect of Vitis vinifera (grape) seed extract and resveratrol. *Phytotherapy Research*.

[89] Rameshrad M., Razavi B. M., Imenshahidi M., Hosseinzadeh H. (2019). Vitis vinifera (grape) seed extract and resveratrol alleviate bisphenol-A-induced metabolic syndrome: biochemical and molecular evidences. *Phytotherapy Research*.

[90] Wang J., Ho L., Zhao W. (2010). Grape-derived polyphenolics prevent A*β* oligomerization and attenuate cognitive deterioration in a mouse model of Alzheimer’s disease. *Journal of Neuroscience*.

[91] Carvalho F. B., Gutierres J. M., Bohnert C. (2015). Anthocyanins suppress the secretion of proinflammatory mediators and oxidative stress, and restore ion pump activities in demyelination. *The Journal of Nutritional Biochemistry*.

[92] Amen Y., Sherif A. E., Shawky N. M., Abdelrahman R. S., Wink M., Sobeh M. (2020). Grape-leaf extract attenuates alcohol-induced liver injury via interference with NF-*κ*B signaling pathway. *Biomolecules*.

[93] Delmas D., Lancon A., Colin D., Jannin B., Latruffe N. (2006). Resveratrol as a chemopreventive agent: a promising molecule for fighting cancer. *Current Drug Targets*.

[94] Gao X., Xu Y. X., Janakiraman N., Chapman R. A., Gautam S. C. (2001). Immunomodulatory activity of resveratrol: suppression of lymphocyte proliferation, development of cell-mediated cytotoxicity, and cytokine production11Abbreviations: CTLs, cytotoxic T lymphocytes; LAK cells, lymphokine activated killer cells; IL-2, interleukin-2; IFN-*γ*, interferon-gamma; TNF-*α*, tumor necrosis factor-*α* NF-*κ*B, nuclear factor kappa B; Con A, concanavalin A; HBSS, Hanks’ balanced salt solution; DTT, dithiothreitol; PMSF, phenylmethylsulfonyl fluoride; RT-PCR, reverse transcription-polymerase chain reaction; LPS, lipopolysaccharide; and EMSA, electrophoretic mobility shift assay. *Biochemical Pharmacology*.

[95] Candelario-Jalil E., de Oliveira A. C. P., Gräf S. (2007). Resveratrol potently reduces prostaglandin E2 production and free radical formation in lipopolysaccharide-activated primary rat microglia. *Journal of Neuroinflammation*.

[96] Bi X. L., Yang J. Y., Dong Y. X. (2005). Resveratrol inhibits nitric oxide and TNF-*α* production by lipopolysaccharide-activated microglia. *International Immunopharmacology*.

[97] Jin F., Wu Q., Lu Y.-F., Gong Q.-H., Shi J.-S. (2008). Neuroprotective effect of resveratrol on 6-OHDA-induced Parkinson’s disease in rats. *European Journal of Pharmacology*.

[98] Zhang F., Shi J.-S., Zhou H., Wilson B., Hong J.-S., Gao H.-M. (2010). Resveratrol protects dopamine neurons against lipopolysaccharide-induced neurotoxicity through its anti-inflammatory actions. *Molecular Pharmacology*.

[99] Sharma M., Gupta Y. K. (2002). Chronic treatment with trans resveratrol prevents intracerebroventricular streptozotocin induced cognitive impairment and oxidative stress in rats. *Life Sciences*.

[100] Khan M. M., Ahmad A., Ishrat T. (2010). Resveratrol attenuates 6-hydroxydopamine-induced oxidative damage and dopamine depletion in rat model of Parkinson’s disease. *Brain Research*.

[101] Tabeshpour J., Mehri S., Shaebani Behbahani F., Hosseinzadeh H. (2018). Protective effects of Vitis vinifera (grapes) and one of its biologically active constituents, resveratrol, against natural and chemical toxicities: a comprehensive review. *Phytotherapy Research*.

[102] Wu Y., Li X., Zhu J. X. (2011). Resveratrol-activated AMPK/SIRT1/autophagy in cellular models of Parkinson’s disease. *Neurosignals*.

[103] Jeong J.-K., Moon M.-H., Bae B.-C. (2012). Autophagy induced by resveratrol prevents human prion protein-mediated neurotoxicity. *Neuroscience Research*.

[104] Zhang F., Lu Y.-F., Wu Q., Liu J., Shi J.-S. (2012). Resveratrol promotes neurotrophic factor release from astroglia. *Experimental Biology and Medicine*.

[105] Bureau G., Longpré F., Martinoli M.-G. (2008). Resveratrol and quercetin, two natural polyphenols, reduce apoptotic neuronal cell death induced by neuroinflammation. *Journal of Neuroscience Research*.

[106] Blanchet J., Longpré F., Bureau G. (2008). Resveratrol, a red wine polyphenol, protects dopaminergic neurons in MPTP-treated mice. *Progress in Neuro-Psychopharmacology and Biological Psychiatry*.

[107] Xu Y., Wang Z., You W. (2010). Antidepressant-like effect of trans-resveratrol: involvement of serotonin and noradrenaline system. *European Neuropsychopharmacology*.

[108] Hurley L. L., Akinfiresoye L., Kalejaiye O., Tizabi Y. (2014). Antidepressant effects of resveratrol in an animal model of depression. *Behavioural Brain Research*.

[109] Ali S. H., Madhana R. M., Athiraa K. V. (2015). Resveratrol ameliorates depressive-like behavior in repeated corticosterone-induced depression in mice. *Steroids*.

[110] Liu D., Xie K., Yang X. (2014). Resveratrol reverses the effects of chronic unpredictable mild stress on behavior, serum corticosterone levels and BDNF expression in rats. *Behavioural Brain Research*.

[111] Dang H., Chen Y., Liu X. (2009). Antidepressant effects of ginseng total saponins in the forced swimming test and chronic mild stress models of depression. *Progress in Neuro-Psychopharmacology and Biological Psychiatry*.

[112] Duman R. S. (2002). Pathophysiology of depression: the concept of synaptic plasticity. *European Psychiatry*.

[113] Irie Y., Itokazu N., Anjiki N., Ishige A., Watanabe K., Keung W. M. (2004). Eugenol exhibits antidepressant-like activity in mice and induces expression of metallothionein-III in the hippocampus. *Brain Research*.

[114] Hackett M. L., Pickles K. (2014). Part I: frequency of depression after stroke: an updated systematic review and meta-analysis of observational studies. *International Journal of Stroke*.

[115] Pang C., Cao L., Wu F. (2015). The effect of trans-resveratrol on post-stroke depression via regulation of hypothalamus-pituitary-adrenal axis. *Neuropharmacology*.

[116] Turner T., Burri B. (2013). Potential nutritional benefits of current citrus consumption. *Agriculture*.

[117] Cui Y., Wu J., Jung S.-C. (2010). Anti-neuroinflammatory activity of nobiletin on suppression of microglial activation. *Biological & Pharmaceutical Bulletin*.

[118] Ho S.-C., Kuo C.-T. (2014). Hesperidin, nobiletin, and tangeretin are collectively responsible for the anti-neuroinflammatory capacity of tangerine peel (Citri reticulatae pericarpium). *Food and Chemical Toxicology*.

[119] Nakajima A., Yamakuni T., Haraguchi M. (2007). Nobiletin, a citrus flavonoid that improves memory impairment, rescues bulbectomy-induced cholinergic neurodegeneration in mice. *Journal of Pharmacological Sciences*.

[120] Onozuka H., Nakajima A., Matsuzaki K. (2008). Nobiletin, a citrus flavonoid, improves memory impairment and A*β* pathology in a transgenic mouse model of Alzheimer’s disease. *Journal of Pharmacology and Experimental Therapeutics*.

[121] Nakajima A., Aoyama Y., Nguyen T.-T. L. (2013). Nobiletin, a citrus flavonoid, ameliorates cognitive impairment, oxidative burden, and hyperphosphorylation of tau in senescence-accelerated mouse. *Behavioural Brain Research*.

[122] Liu Y., Wu H., Nie Y.-c., Chen J.-l., Su W.-w., Li P.-b. (2011). Naringin attenuates acute lung injury in LPS-treated mice by inhibiting NF-*κ*B pathway. *International Immunopharmacology*.

[123] Kim H.-J., Song J. Y., Park H. J., Park H.-K., Yun D. H., Chung J.-H. (2009). Naringin protects against rotenone-induced apoptosis in human neuroblastoma SH-SY5Y cells. *The Korean Journal of Physiology and Pharmacology*.

[124] Kim H. D., Jeong K. H., Jung U. J., Kim S. R. (2016). Naringin treatment induces neuroprotective effects in a mouse model of Parkinson’s disease in vivo, but not enough to restore the lesioned dopaminergic system. *The Journal of Nutritional Biochemistry*.

[125] Leem E., Nam J. H., Jeon M.-T. (2014). Naringin protects the nigrostriatal dopaminergic projection through induction of GDNF in a neurotoxin model of Parkinson’s disease. *The Journal of Nutritional Biochemistry*.

[126] Golechha M., Sarangal V., Bhatia J., Chaudhry U., Saluja D., Arya D. S. (2014). Naringin ameliorates pentylenetetrazol-induced seizures and associated oxidative stress, inflammation, and cognitive impairment in rats: possible mechanisms of neuroprotection. *Epilepsy & Behavior*.

[127] Kumar A., Prakash A., Dogra S. (2010). Naringin alleviates cognitive impairment, mitochondrial dysfunction and oxidative stress induced by D-galactose in mice. *Food and Chemical Toxicology*.

[128] Raza S. S., Khan M. M., Ahmad A. (2011). Hesperidin ameliorates functional and histological outcome and reduces neuroinflammation in experimental stroke. *Brain Research*.

[129] Kumar B., Gupta S. K., Srinivasan B. P. (2013). Hesperetin rescues retinal oxidative stress, neuroinflammation and apoptosis in diabetic rats. *Microvascular Research*.

[130] Shu Z., Yang B., Zhao H. (2014). Tangeretin exerts anti-neuroinflammatory effects via NF-*κ*B modulation in lipopolysaccharide-stimulated microglial cells. *International Immunopharmacology*.

[131] Genovese S., Epifano F. (2011). Auraptene: a natural biologically active compound with multiple targets. *Current Drug Targets*.

[132] Furukawa Y., Washimi Y. S., Hara R. I. (2020). Citrus auraptene induces expression of brain-derived neurotrophic factor in neuro2a cells. *Molecules*.

[133] Okuyama S., Minami S., Shimada N., Makihata N., Nakajima M., Furukawa Y. (2013). Anti-inflammatory and neuroprotective effects of auraptene, a citrus coumarin, following cerebral global ischemia in mice. *European Journal of Pharmacology*.

[134] Okuyama S., Morita M., Kaji M. (2015). Auraptene acts as an anti-inflammatory agent in the mouse brain. *Molecules*.

[135] Okuyama S., Semba T., Toyoda N. (2016). Auraptene and other prenyloxyphenylpropanoids suppress microglial activation and dopaminergic neuronal cell death in a lipopolysaccharide-induced model of Parkinson’s disease. *International Journal of Molecular Sciences*.

[136] Okuyama S., Yamamoto K., Mori H. (2014). Auraptene in the peels of Citrus kawachiensis (Kawachi Bankan) ameliorateslipopolysaccharide (LPS)-induced inflammation in the mouse brain. *Evidence-Based Complementary & Alternative Medicine*.

[137] Olesen J., Gustavsson A., Svensson M. (2012). The economic cost of brain disorders in Europe. *European Journal of Neurology*.

[138] Wimo A., Jönsson L., Bond J., Prince M., Winblad B. (2013). The worldwide economic impact of dementia 2010. *Alzheimer’s & Dementia*.

[139] FDA (2018). Code of federal regulations title 21. https://www.accessdata.fda.gov/scripts/cdrh/cfdocs/cfcfr/CFRSearch.cfm?fr=101.12.

