# Supplementary material for: Potency of Selected Berries, Grapes, and Citrus Fruit as Neuroprotective Agents
Source: Evid Based Complement Alternat Med. 2020 May 30;2020:3582947. doi: 10.1155/2020/3582947 (PMC7277024; doi:10.1155/2020/3582947)
Supplement: Supplementary Materials — Table 1: summary of the neuroprotective potency of selected fruits. [file 3582947.f1.docx]

| **Fruit type** | **Protective effect against** | **Bioactivity** | **Putative Active Compound** |
| --- | --- | --- | --- |
|  |  |  |  |
| Berries (blueberries, blackberries, raspberries, cranberries, etc.) | Parkinson’s disease (PD) and Alzheimer’s disease (AD), nitric oxide synthase (iNOS) and cyclooxygenase-2 (COX-2), various oxidative stress-related disorders, abnormal acetylcholinesterase activity, and diabetic neuropathy | Immunomodulating properties, neuromodulatory properties | Anthocyanin and proanthocyanin, e.g. cyanidin-3-*O*-glucoside, and ellagic acid |
| Grape (*Vitis vinifera*) | Poststroke depression (PSD) | Natural neurotrophic agent, endogenous antioxidant enzyme activities, weakening abnormal or prolonged inflammatory events, immunomodulatory effects | Anthocyanin, proanthocyanin, and resveratrol (3,4’,5-trihydroxy-*trans*-stilbene). |
| Citrus Fruit (*Citrus* spp.: clementines (*C. clementine*), grapefruits (*C. paradisi*), lemons (*C. limon*), limes (*C. aurantifolia*), oranges (*C. sinensis*), and tangerines (*C. reticulata*)) | Neurological disorders, PD neurotoxicity, bronchial asthma and dyspepsia, diabetic neuropathies. | Antiepileptic activity | Vitamin C, nobiletin, naringin (4’,5,7-trihydroxyflavanone-7-rhamnoglucoside), hesperidin (3’,5,7-trihydroxy-4’-methoxyflavone-7-rhamnoglucoside) |

**Table 1: Summary of the neuroprotective potency of selected fruits**
